# Synthesis, Urease Inhibition, Molecular Docking, and Optical Analysis of a Symmetrical Schiff Base and Its Selected Metal Complexes

**DOI:** 10.3390/molecules29204899

**Published:** 2024-10-16

**Authors:** Samuel Bonne, Muhammad Saleem, Muhammad Hanif, Joseph Najjar, Salahuddin Khan, Muhammad Zeeshan, Tehreem Tahir, Anser Ali, Changrui Lu, Ting Chen

**Affiliations:** 1 College of Biological Science and Medical Engineering, Donghua University, Shanghai 201620, China; 2 Faculty of Medicine, McGill University, Montréal, QC H3A 0G4, Canada; 3 Department of Chemistry, Thal University Bhakkar, Bhakkar 30000, Pakistan; 4 Department of Chemistry, University of Sargodha, Sargodha 40162, Pakistan; 5 Department of Chemistry, GC University Faisalabad, Sub Campus, Layyah 31200, Pakistan; 6 College of Engineering, King Saud University, P.O. Box 800, Riyadh 11421, Saudi Arabia; 7 Department of Physiology and Biochemistry, Cholistan University of Veterinary and Animal Sciences, Bahawalpur 63100, Pakistan; 8 Department of Biological Sciences, Mirpur University of Science and Technology (MUST), Mirpur 10250, AJK, Pakistan

**Keywords:** Schiff base, urease inhibition, optical analysis, molecular docking, enzyme kinetics

## Abstract

Designing and developing small organic molecules for use as urease inhibitors is challenging due to the need for ecosystem sustainability and the requirement to prevent health risks related to the human stomach and urinary tract. Moreover, imaging analysis is widely utilized for tracking infections in intracellular and in vivo systems, which requires drug molecules with emissive potential, specifically in the low-energy region. This study comprises the synthesis of a Schiff base ligand and its selected transition metals to evaluate their UV/fluorescence properties, inhibitory activity against urease, and molecular docking. Screening of the symmetrical cage-like ligand and its metal complexes with various eco-friendly transition metals revealed significant urease inhibition potential. The IC_50_ value of the ligand for urease inhibition was 21.80 ± 1.88 µM, comparable to that of thiourea. Notably, upon coordination with transition metals, the ligand–nickel and ligand–copper complexes exhibited even greater potency than the reference compound, with IC_50_ values of 11.8 ± 1.14 and 9.31 ± 1.31 µM, respectively. The ligand–cobalt complex exhibited an enzyme inhibitory potential comparable with thiourea, while the zinc and iron complexes demonstrated the least activity, which might be due to weaker interactions with the investigated protein. Meanwhile, all the metal complexes demonstrated a pronounced optical response, which could be utilized for fluorescence-guided targeted drug delivery applications in the future. Molecular docking analysis and IC_50_ values from in vitro urease inhibition screening showed a trend of increasing activity from compounds **7d** to **7c** to **7b**. Enzyme kinetics studies using the Lineweaver–Burk plot indicated mixed-type inhibition against **7c** and non-competitive inhibition against **7d**.

## 1. Introduction

**Scheme 1:**
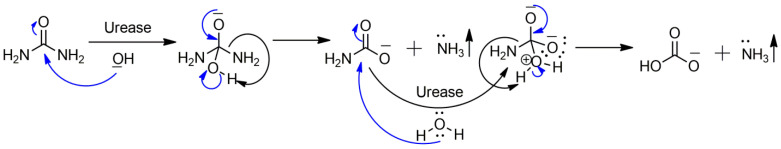
Mechanism of urea hydrolysis by urease.

The urease enzyme (EC 3.5.1.5) plays a crucial role in fungi (*Aspergillus fumigatus*), bacteria (*Helicobacter pylori* (*H. pylori*) and *Proteus mirabilis* (*P. mirabilis*)), and plants (*Jack Bean*). Notably, it was the first enzyme to be crystallized in 1926 [1,2,3]. Urease facilitates and catalyzes urea hydrolysis to carbamate and ammonia, which further decomposes to ammonia and bicarbonate, contributing to the nitrogen cycle (Scheme 1). However, excessive urease activity can lead to high levels of ammonia, resulting in ammonia toxicity in the atmosphere and soil nitrogen deficiency, which deprives plants of nutrients. During urea fertilization in the soil, increased urease activity converts a significant amount of urea into gaseous ammonia, exacerbating ammonia toxicity and causing economic losses and nutrient depletion in plants [4,5]. In humans, the *H. pylori* bacteria produces urease that hydrolyzes urea into ammonia, prompting the stomach to secrete large amounts of acid that damage its mucous lining, leading to gastric or peptic ulcers. Ammonia is also toxic to various gastric cells, creating a conducive environment for *H. pylori*, which further leads to gastric ulcerations and stomach cancers [6,7]. Another urease-positive bacterium, *Proteus mirabilis*, builds up biofilms over as well as around the surface of the catheter. The bacterium utilizes urease as the source of nitrogen through urea hydrolysis. This urease catalysis reaction increases urine pH, promoting crystal formation in the *Proteus mirabilis* biofilm connected to the catheter and causing obstruction in urine flow towards the drainage bag [8,9]. To address urease-related issues in both plants and animals, designing and developing urease inhibitors is essential. Such inhibitors should ideally have dual applicability, targeting bacterial strains and inhibiting both bacterial and fungal urease.

In recent years, several molecular scaffolds have been reported as urease inhibitors, including hydrazine derivatives [10], isoniazid [11], pyrazoline [12], sulfadiazine [13], thiourea [14], and thioxothiazolidinyl [15]. Among these, nitrogen-containing heterocyclic azole derivatives have garnered significant attention for addressing stomach-related issues. Notably, commercial azole derivatives, such as the omeprazole family, are proton pump inhibitors used to reduce stomach acid concentrations. Azole derivatives, including thiazole [16], thiadiazole [17], thiazolidine [18], pyrazole [19], and triazole [20], and fused azole derivatives, such as triazolothiadiazole [21,22], can also suppress the acidic flux resulting from bacterial urease inhibition activity in the stomach. In addition to these heterocyclic scaffolds, numerous reports have highlighted the potential of heterocyclic Schiff base complexes [23], which exhibit significant urease inhibition activity. These include copper [24], cobalt [25], gold [26], vanadium [27], silver [28], nickel [29], cadmium [30], and cobalt complexes [31]. Here, we studied the synthesis of a symmetrical Schiff base ligand with a triazole moiety and its metal complexes for urease inhibition activity and obtained appreciable results. Meanwhile, all the metal complexes demonstrated a pronounced optical response, which could be utilized for fluorescence-guided targeted drug delivery applications in the future [32,33,34].

## 2. Results and Discussion

### 2.1. Synthesis of Ligand and Its Metal Complexes

The ligand synthesis was carried out by starting from *para*-methoxybenzoic acid, which was initially esterified to the ethyl ester by refluxing with ethyl alcohol and a small quantity of sulfuric acid. The initial assessment was conducted by TLC employing aluminum precoated silica gel plates and chloroform/methanol (9:1) and hexane/ethylacetate (8:2) as eluents. The acid appeared at the bottom of TLC plates in the 20% hexane/ethylacetate solution, while the esters appeared with a reasonable Rf value. The morphological variations from white solids to oily liquids took place during the esterification reaction. The confirmation of esterification was achieved through FT-IR spectral analysis, indicated by the reduction of the broad acid–hydroxyl signal peak with values ranging from 3400 to 2500 cm^−1^. The purpose of the ester formation was to transform the poor leaving group into the good leaving group for the upcoming hydrazide formation reaction, where hydrazine acted as a nucleophile and removed the ethoxy via a substitution reaction. This substitution was carried out by refluxing the ester of *p*-methoxybenzoic acid with hydrazine hydrate for around 12 h, which afforded hydrazides as white solids on removing the solvent under reduced pressure. The estimation of the hydrazide formation was carried out by a morphological change assessment from oily material into white solids, which then recrystallized to needle-like crystals with a melting point of 138 °C. The vibrational spectroscopic analysis led to the shortening of the carbonyl stretching vibrational signal, along with the appearance of sharp amino peaks of the amide moiety in the range of 3342 to 3158 cm^−1^. The next step was nucleophilic substitution of the hydrazide with the amino isocyanate to produce the substituted urea derivative by following substitution through the tetrahedral pathway. After the complete consumption of the reacting species, the materials were subjected to symmetrical pyridine dialdehyde to afford an imine derivative, which, upon refluxing with triethylamine in the tetrahydrofuran, underwent cyclization via dehydration to produce yellow precipitates of the target molecule 6. The characterization of 6 was carried out by the appearance, melting point determination, and FT-IR and NMR analyses. The vibrational spectroscopic analysis of ligand 6 exhibited a slightly broad peak in the range of 3651 to 3520 due to amino stretching vibrations. Amino signals are normally sharp, so the reason for the slight broadness was due to the involvement of amino protons in the tautomerism in the triazole ring of ligand 6, which induced a partial hydroxyl character as well. The aromatic carbon-to-hydrogen bond vibrated in the range of 3180 to 2840 cm^−1^, while the high-intensity peak at 1700 cm^−1^ was due to the triazole ring carbonyl group. The ring -C=N bond appeared at 1657 and 1609 cm^−1^. Proton NMR analysis revealed a highly de-shielded peak due to the triazole N-H. The resonance of the Schiff base protons was observed at 10.18 ppm as a two-proton singlet. The pyridine skeleton showed two distinct de-shielded signals: one ranged from 8.256 to 8.249 ppm (doublet, 2H, *J* = 2.8 Hz) and the other from 8.198 to 8.176 ppm (triplet, 1H, *J* = 8.8 Hz). The protons from the anisole moiety exhibited two sets of multiplets with a multiplicity of 4 for each signal. A singlet signal in the aliphatic region at 3.83 ppm with the integration of six was assigned to the methoxy group. The carbon NMR exhibited one aliphatic band at 55.86 ppm due to the methoxy group, while the rest of the signals were observed in the aromatic region.

**Scheme 2:**
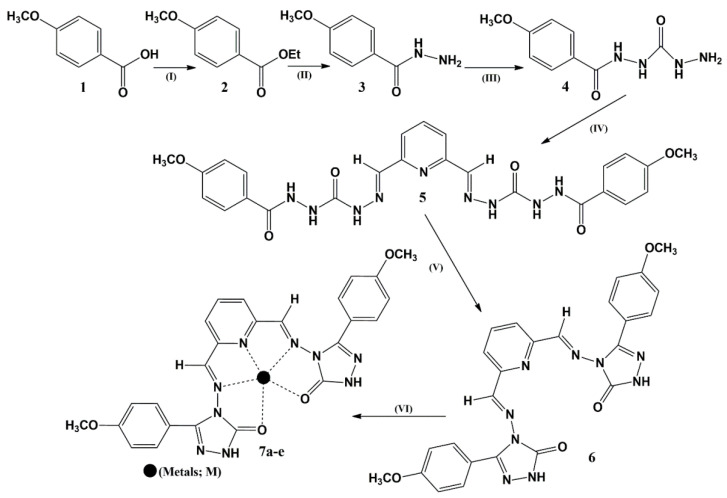
Synthetic pathway adopted for the accomplishment of target molecules: (I) C_2_H_5_OH; H_2_SO_4_; reflux; overnight. (II) N_2_H_4_.H_2_O; C_2_H_5_OH; reflux; overnight. (III) Aminoisocyanate; C_2_H_5_OH; reflux; 4–5 h. (IV) Pyridine-2,6-dicarbaldehyde; THF; reflux; overnight. (V) In situ; reflux; 12–14 h. (IV) Metal salt solution; C_2_H_5_OH; reflux; 3–4 h; **7a** [ligand–Fe(II)]; **7b** [ligand–Co(II)]; **7c** [ligand–Ni(II)]; **7d** [ligand–Cu(II)]; **7e** [ligand–Zn(II)].

The synthesized metal complexes **7a**–**e** were initially analyzed by colorimetric variations for the appearance from yellow to reddish, greenish, brick red, mustard yellow, and gray for the iron, cobalt, nickel, copper, and zinc complexes. Ligand exhibited a melting point of 181–183 degrees Celsius, which was observed to be enhanced after complexation, with a range of 218–220, 231–233, 234–236, 228–230, and 216–218 for the iron, cobalt, nickel, copper, and zinc complexes, respectively. Further characterization was carried out by UV, fluorescence, and FT-IR analyses. The complexation reaction caused slight variations in the lactam carbonyl stretching vibration with values of 1700, 1675, 1698, 1709, and 1712 cm^−1^ for the iron, cobalt, nickel, copper, and zinc complexes, respectively, reflecting the involvement of these skeletons in the complexation reactions (Table 1). Another prominent change was in the stretching vibration of the iminic group after complexation, with the overall trend of increase in the vibrational frequency. Meanwhile, noticeable spectral shifting was observed for the triazole -C=N stretching vibration, which was originally at 1598 and 1587 cm^−1^, for the ligand while undergoing an increase in the wavenumber of the peak, indicating the involvement of the triazole moiety in the complexation with the metal ions. The stretching vibration values of the iminic group in the triazole occur at slightly higher frequencies due to the tautomeric form of the triazole molecule, which imparts a partial single bond character. Conversely, the imine group adjacent to the pyridine structure will vibrate at slightly lower frequencies compared to the imine in the triazole. The schematic representation of the synthetic pathway for the accomplishment of the target molecule and its metal complexes is shown in Scheme 2.

### 2.2. Optical Analysis

#### 2.2.1. UV–Visible Absorption Analysis

**Figure 1:**
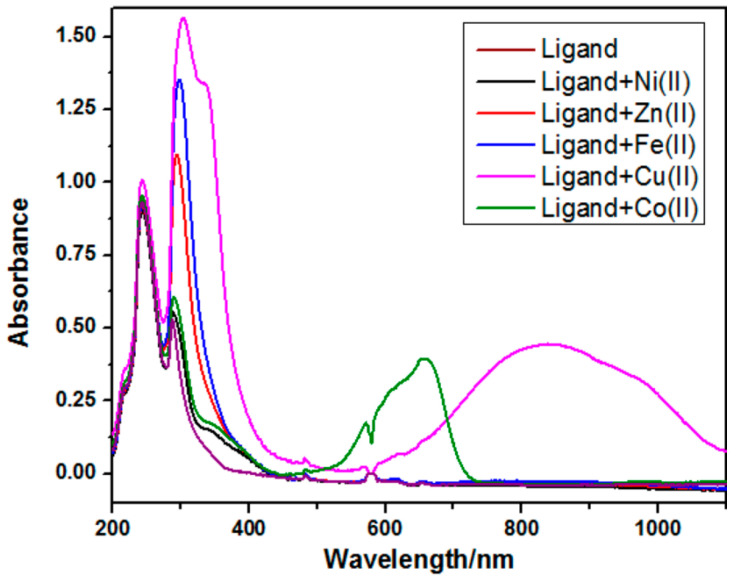
The absorption spectra of the ligand and its corresponding metal complexes.

The targeted ligands are symmetrical, with the two triazole moieties on both termini exhibiting a total of five heteroatoms in a cage-like pattern, which are plausible configurations to trap and chelate the transition metals. The ligand metal complexation reaction was carried out by equimolar stoichiometric combination in the appropriate solvent at boiling temperature, which initially afforded colorimetric variations in the reaction mixture with green, gray, red, mustard yellow, and brick red colors for the cobalt, zinc, iron, copper, and nickel complexes, respectively. These colored complexes exhibited a fascinating pattern of signal in the UV–visible region of the electromagnetic range. Briefly, the two absorption bands at 241 and 291 nm by the ligand were due to the two different types of electronic transitions by the pi and non-bonding electrons. The first signal with the higher absorbance intensity was assigned to the absorbance due to the *pi*-electrons system, while the second, slightly less intense peak was assigned to the absorbance coming from lone pairs of electrons. The copper and cobalt complexes exhibited peculiar behavior with the appearance of two signals in the range of 200–500 nm and one signal for each complex in the near-infrared regions. These high-intensity near-infrared region signals for cobalt and copper were due to the ease of electronic flow from the ligand non-bonding electrons to the metal conduction bands, and these facile electronic flows reduced the overall transition energy. Meanwhile, the absorption signal due to both cobalt and copper exhibited shoulder signals as well, with the peak at 336 nm in the case of copper and at 349 and 571 nm for the cobalt complex. The Ni complex showed absorbance at 243 and 291 nm with the shoulder at 342 nm. The zinc and iron complexes exhibited well-defined electronic transitions at 241 and 296 nm and 242 and 299 nm, respectively (Figure 1, Table 2).

**Figure 2:**
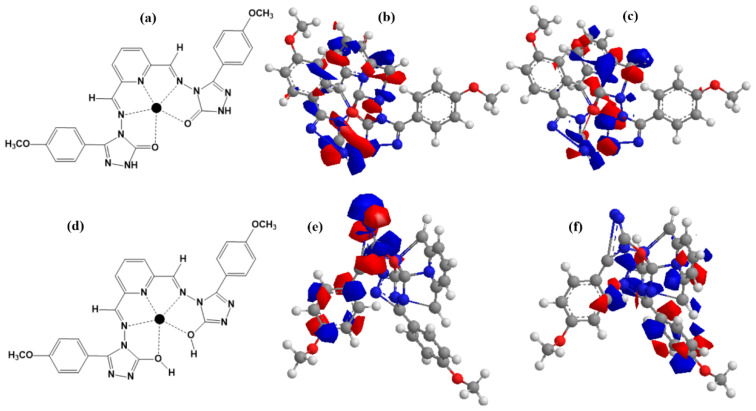
Chemical structure of the ligand’s complex tautomeric forms and their Huckel structures; (**a**,**d**) are chemical structures of two tautomers; (**b**,**e**) are HOMO; and (**c**,**f**) are the LUMO Huckel representations.

To evaluate the peculiarity in the electronic transition in the case of cobalt and copper, computational analysis was carried out by drawing Huckel surfaces of the two different possible tautomeric forms of the ligand, as shown in Figure 2. The electron density was spread over the broader regions, including the heterocyclic skeleton to the anisol skeleton in the case of the enol isomeric form, while the electron density was limited to the iminic skeletons in the case of the keto isomeric form of the ligand–metal complexes. The distribution of the electronic density over the broader regions in the case of the enol form may cause facile and low-energy electronic transitions. From these observations, it was derived that the enol form of the ligand facilitates complexation towards copper and cobalt, while the keto form makes complexation with the rest of the metals.

#### 2.2.2. Solvent Tolerance

The ability of the ligand–metal complexes to withstand varieties of solvents was determined using four different solvents: tetrahydrofuran, ethanol, dimethyl sulfoxide, and acetonitrile. In all these solvents, the ligand and its metal complexes exhibited appreciable absorbance features with good molar absorption and well-defined signals. These optical features of the synthesized metal complexes over a versatile range of media might be prominent features for their utility in drug discovery, bioimaging, and sensors. The electronic transitions observed in the ligand and its metal complexes across various organic media are summarized in Table 3.

#### 2.2.3. Fluorescence Analysis

**Figure 3:**
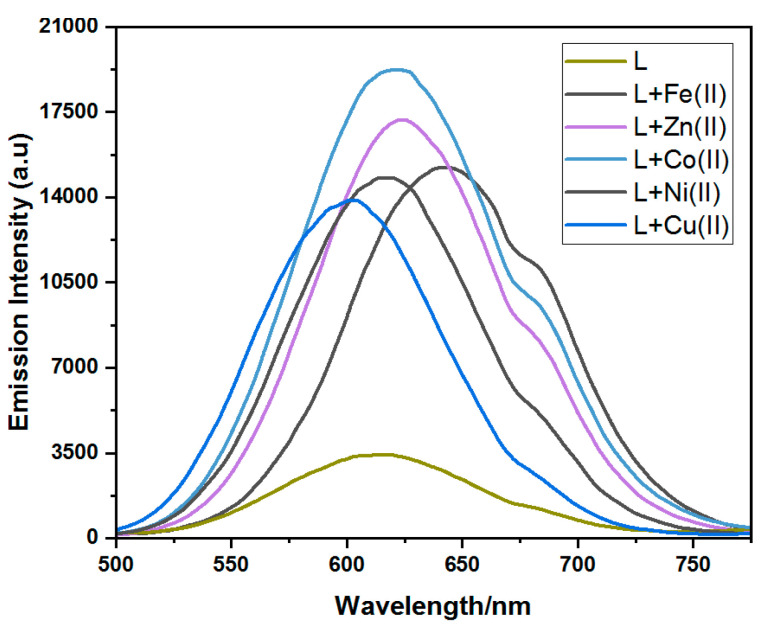
Fluorescence spectra related to the ligand and its respective complexes.

The ligand was weakly emissive at low concentrations while exhibiting a considerable emission signal at the 100 µM concentration level, and this concentration was utilized to record emission spectra of the ligand and its corresponding complexes. The ligand exhibited a broad fluorescence band at 614 nm with a signal height of 3482 a.u. upon excitation at 350 nm. Only the ligand–copper complex showed enhanced emission, with an emission intensity of 13,863 a.u. at 601 nm alongside a 13 nm blue shift from the ligand emission bands. All other complexes showed a bathochromic shift in the emission spectra at 643, 623, 621, and 617 nm and emission intensities of 15,253, 17,185, 19,270, and 14,867 a.u. for the iron, zinc, cobalt, and nickel complexes, respectively. The maximum redshift of 29 nm was observed for the ligand–iron complex, and the rest of the red-shifted values were 9, 7, and 3 nm for the L-zinc, L-cobalt, and L-nickel complexes, respectively (Figure 3). The quantum yield values were in the range of 0.09, 0.14, 0.18, 0.15, and 0.17 for the iron, cobalt, nickel, copper, and zinc complexes, respectively (Table 4).

### 2.3. Bioevaluation

#### 2.3.1. Urease Inhibition Activity

Recent literature contains several reports highlighting the urease inhibition potential of substituted triazole scaffolds (Table 5). Mahdavi and colleagues described two slightly different substituted triazole skeletons exhibiting significant activity, with IC_50_ values in the range of 76.53 ± 0.46 to 08.10 ± 0.17 µM [35] and 184.43 ± 2.16 to 22.81 ± 0.05 µM against urease [36], compared to the reference compounds thiourea and hydroxyurea. Another 1,2,4-triazole scaffold, HL, was reported by Xu and colleagues [37] with urease inhibition values of 15.094 ± 2.218 µM. This inhibition activity was further enhanced following complexation reactions, yielding IC_50_ of 4.052 ± 0.693 and 6.868 ± 1.006 µM for Zn^2+^ and Fe^2+^ complexes. Moreover, previous studies have shown enhanced urease inhibition activity of triazole-based ligands upon forming cobalt and nickel complexes [38,39].

Herein, we present the synthesis of a ligand symmetrically connected to two triazole skeletons. This design aims to incorporate two inhibition units within a single molecule and facilitate its complexation with various eco-friendly transition metals. The triazole ligand and its corresponding complexes were screened for enzymatic activity, demonstrating significant results regarding the urease inhibition assay. The symmetrical triazole ligand had an IC_50_ value of 21.80 ± 1.88, around that of thiourea. Interestingly, after ligation to the transition metals, the activities of the ligand–nickel and ligand–copper complexes were found to be more potent than those of the reference, with IC_50_ values in the range of 11.8 ± 1.14 and 09.31 ± 1.31 µM, respectively. The ligand–cobalt complex showed urease inhibition potential almost that of thiourea. The least activity was observed against the zinc and iron complexes, which might be due to less interaction of these complexes with the tested protein (Table 6).

#### 2.3.2. Mechanism Underlying Compounds **7c** and **7d**’s Inhibitory Action

**Figure 4:**
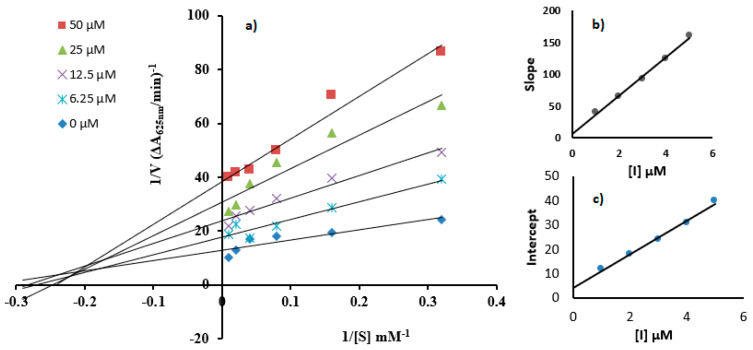
(**a**) Lineweaver–Burk plots showing urease inhibition using urea as substrate with concentrations of 3.12, 6.25, 12.5, 25, 50, and 100 mM and inhibitor as compound **7c** with concentrations of 0, 6.25, 12.5, 25, and 50 µM. Insets represent graphical representations of slope and intercept vs. inhibitor concentration (inset (**b**,**c**)).

**Figure 5:**
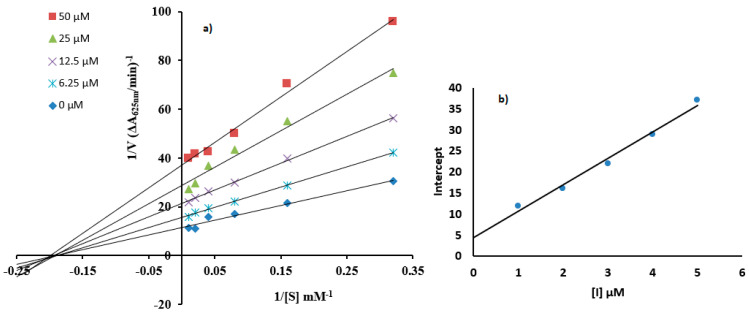
(**a**) Lineweaver–Burk plots showing urease inhibition when compound (**7d**) is present with concentrations of 0, 6.25, 12.5, 25, and 50 µM with the urea as the substrate with concentrations of 3.12, 6.25, 12.5, 25, 50, and 100 mM. (**b**) Representation of the graph of intercept verses **7d** concentrations.

Enzyme kinetics against urease was evaluated to obtain better insight towards the inhibitor mechanism underlying the tested compounds using Lineweaver–Burk plot of inverses of V and S against variable concentrations of **7c** and **7d**. The results produced a succession of linear responses, as illustrated in Figure 4 and Figure 5. In addition, secondary replots were used to figure out the EI (*Ki*) and ESI (*Ki’)* dissociation constant values to better obtain sagaciousness. Upon an increase in the urea concentration, the families of straight lines produced intersected within the second quadrant in the case of molecule **7c**, while a similar response was afforded on the *x*-axis for compound **7d**. The increasing concentration of **7c** exhibited an increase in the Michaelise–Menten constant (K_m_) with a corresponding decline in reaction velocity (V_max_). This response reflected the mixed-type inhibition by the tested compound toward urea, affording *Ki* values of 0.20 µM and *Ki’* values of 0.60 µM, as seen in Figure 4b,c [40]. Meanwhile, an increase in the concentration of **7d** caused a drop in the reaction velocity (V_max_) with constant Km. These observations reflect the non-competitive inhibitory response relative to the *Ki’* value of 0.80 µM (Figure 5).

### 2.4. Molecular Docking

**Figure 6:**
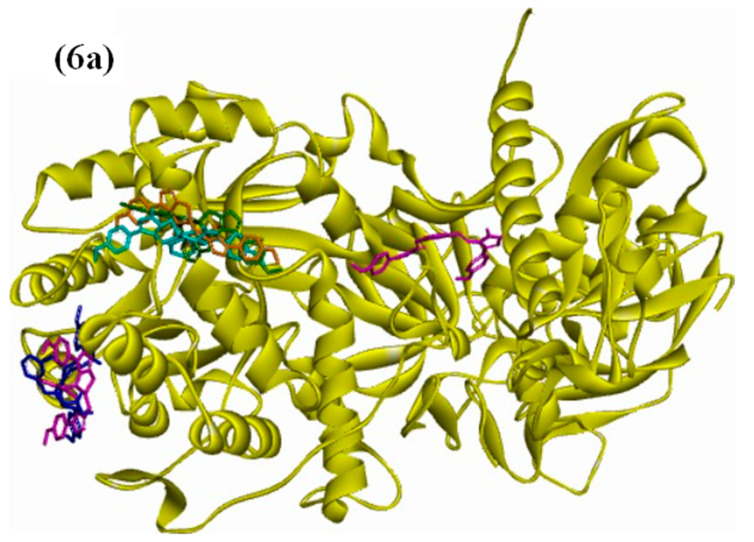

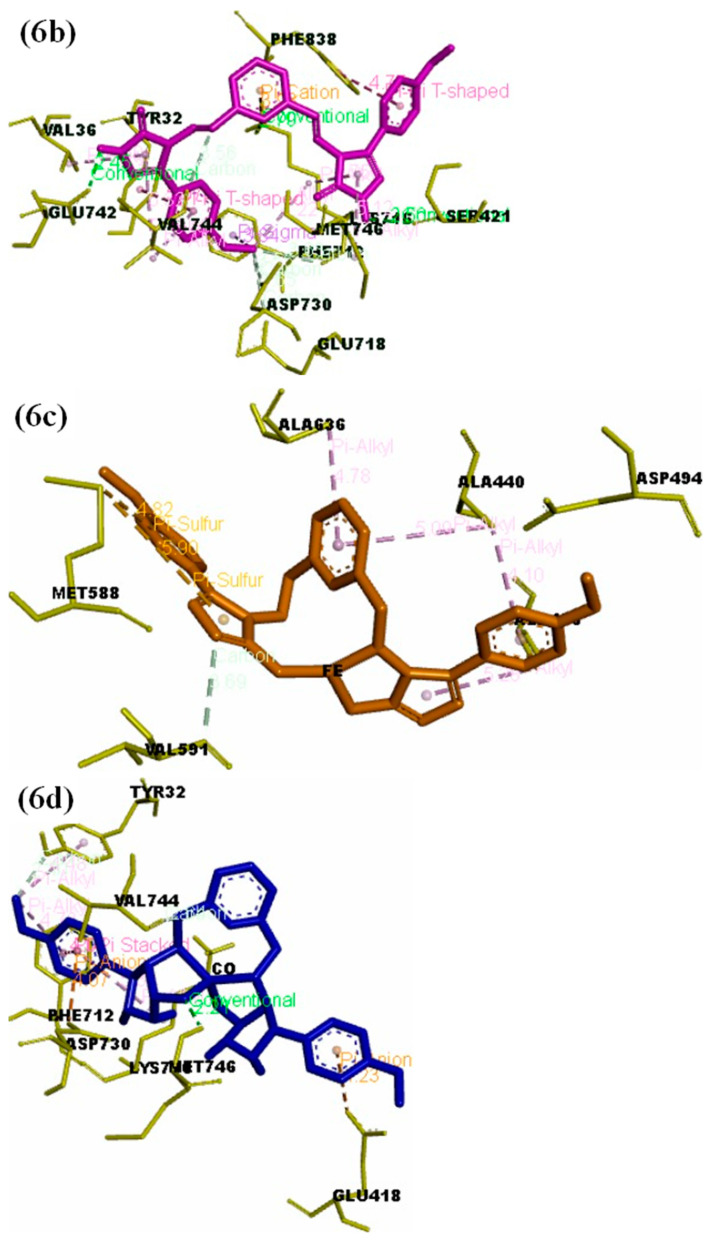

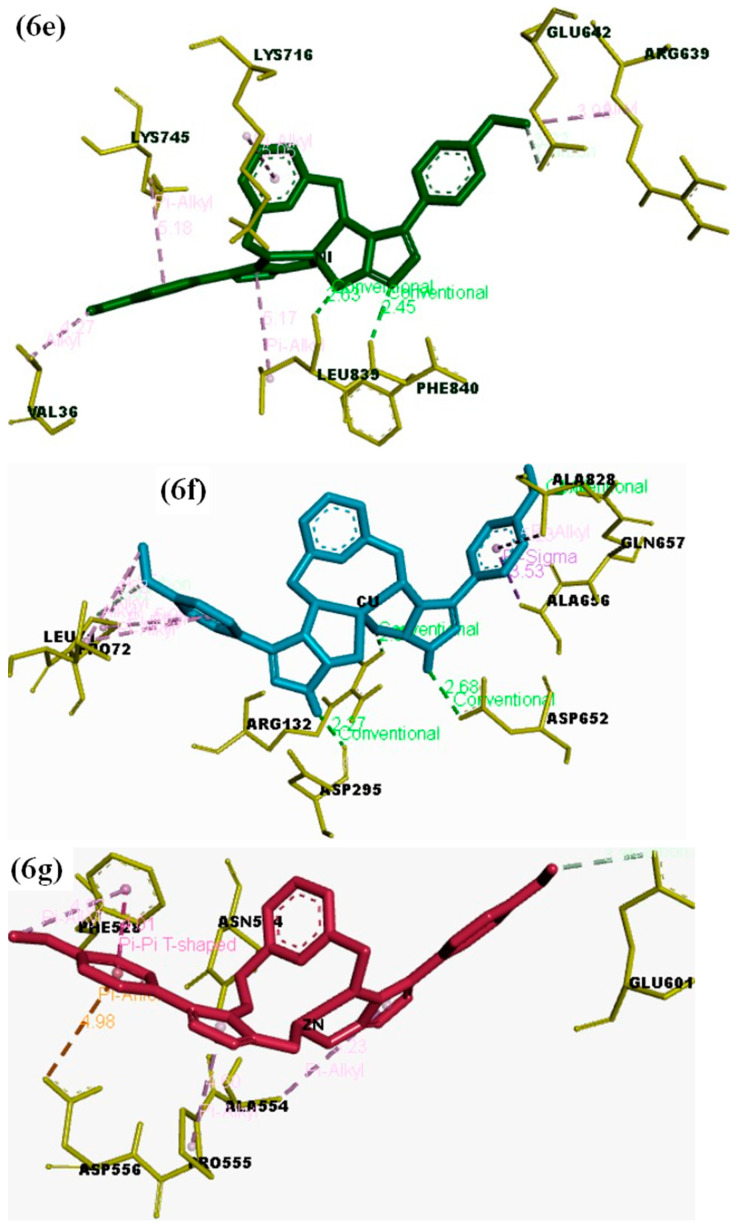
(**a**) Docked complex of the objective Jack Bean urease protein (PDB: 3LA4) with compounds **6** and **7a**–**e**; (**b**) 3D representation showing the key interacting groups between Jack Bean urease (PDB: 3LA4) and compound **6**; (**c**) **7a**; (**d**) **7b**; (**e**) **7c**; (**f**) **7d**; (**g**) **7e**.

The structure–activity relationship (SAR) between the target protein (3LA4) and the test compounds [(**6**, **7a**, **7b**, **7c**, **7d**, and **7e**); Figure 6a–g] were investigated through docking analysis [41,42]. Specifically, 90% residue was found in the preferred region using the Ramachandran plot, with excellent accuracy of both the ϕ and ψ angles of the tested protein’s coordinates (Figure S1; Supporting Information). In silico analysis revealed that the most active compound, **7d**, among the series made a stable docked complex with the target protein 3LA4 with a docking score of −10.4 kcal/mol. The SAR showed that both methyl groups formed active hydrogen bonds with the Glu657 and Pro72 residues. The benzene rings were found to have hydrophobic interactions (π–alkyl and π–sigma) with the residues Ala656, Ala228, Pro72, and Leu72. Moreover, the triazole rings of the compound **7d** also exhibited stable hydrogen bonding interactions with Asp652, Arg132, and Asp295 (Figure 6d). SAR investigations of **7b** exhibited stable hydrogen bonding with the residues of Val744, Tyr32, and Phe712 through the methyl substituent. Interactions such as π–π stacking and π–alkyl bonding were observed between the benzene rings of compound **7b** and the residues Phe712, Asp730, Glu418, and Lys716. The oxygen atom of compound **7b**, which was also coordinated with Co^2+^, displayed active *H*-bonding with the residue Met746 (Figure 6b). Compound **6** also exhibited a wide array of interactions with its neighboring residues, and most of them were conventional hydrogen bonds. The key interacting groups were triazole and pyridine of compound **6** with residues Lys716, Ser421, and Glu742. However, the terminal benzene rings participated in hydrophobic interactions (π–alkyl and π–sigma) with Phe838, Tyr32, Phe712, and Val744 (Figure 6). Regarding compound **7c**, the terminal methyl groups were found to engage with the active site residues Glu642 and Val36 through hydrogen bonding. The benzene and pyridine rings of **7c** were found to make hydrophobic (π–alkyl) interactions with Lys745 and Lys716. Additionally, *H*-bonding associations were detected among the triazole rings of compound **7c** and the residues Leu839 and Phe840 (Figure 6c). In the case of compounds **7a** and **7e**, few hydrophobic interactions were observed between pyridine and benzene rings with the residues Ala636, Ala440, Ala436, Met588, Ala554, Asp556, and Pro555. The binding energies of **7a** (Figure 6c) and **7e** (Figure 6g) suggested that both of these compounds sit closely with each other in the binding pocket of the enzyme (3LA4). Overall, the binding energies and IC_50_ values exhibited the following trend from lower to higher: **7d** < **7c** < **6** < **7b** < **7a** < **7e** (Table 6).

## 3. Experimental

### 3.1. Substrate and Reagents

Here, *p*-methoxybenzoic acid, 2,6-pyridinedicarbaldehyde, POCl_3_, carbamoyl azide, N_2_H_4_ (80%), CH_3_COOH (glacial), triethylamine, KOH, NaOH, NaHCO_3_, molecular sieves, magnesium turning, and iodine pellets from Aldrich were utilized. Analytical-grade chloride salts of iron, copper, zinc, cobalt, and nickel were utilized throughout the experimentation and purchased from Aldrich, Darmstadt, Germany. The solvents, including ethyl alcohol, acetonitrile, dimethylsulfoxide, tetrahydrofuran, ethylacetate, *n*-hexane, chloroform and methylalcohol, were purchased from Aldrich and Samchun, Pyeongtaek-si, Republic of Korea.

### 3.2. Instrumentations

Aluminum-precoated thin-layer chromatographic plates made by Merck, Darmstadt, Germany, were employed. Kieselgel 60 F_254_ from Fisher Scientific, Waltham, MA, USA, was used. Drying of products was performed using a vacuum desiccator before spectroscopic analysis. Vacuum filtration assemblies with vacuum pumps and Buckner/sintered glass funnels from BIOBASE, Shandong, China were employed to filter the products during the experimentation. The vibrational spectroscopic analysis of the products was carried out using the Japan-made Shimadzu FTIR–8400S spectrometer (Kyoto, Japan). A Bruker Avance (Ettlingen, Germany) 400 MHz NMR instrument was employed to record the proton and carbon NMR spectra. The Scinco (Seoul, Republic of Korea) absorption and emission spectrophotometer was utilized for optical analysis.

### 3.3. Characterization of Schiff Base Ligand ***6***

*p*-methoxybenzoic acid (1; 3 g) was esterified by refluxing in ethanol (30 mL) and in the presence of a catalytic amount of sulfuric acid for 4–6 h. *p*-methoxybenzoate (2) was converted into the corresponding 4-methoxybenzohydrazide (3) by refluxing in hydrazine hydrate (5 mL) in methanol (30 mL) following the procedure in the literature [43,44,45]. This hydrazide (3; 1 molar; 4.98 g) was reacted with aminoisocyanate (1 molar; 1.74 g) in ethanol (30 mL) under reflux for 4–5 h to afford urea molecule (4), which was then reacted with pyridine-2,6-dicarbaldehyde (12 mL) in tetrahydrofuran under reflux for 24 h to afford target molecule **6** as yellow powder and purified with column chromatography using dichloromethane as eluent with polarity adjustment using ethanol (Supporting Information Figures S2–S4).

#### 3.3.1. 4,4′-((1*E*,1′*E*)-(Pyridine-2,6-diylbis(methanylylidene))bis(azanylylidene))bis(3-(4-methoxyphenyl)-1*H*-1,2,4-triazol-5(4*H*)-one) (**6**)

Yellow powder; yield: 58%; 2.4 g; mp: 181–183 °C; R*_f_*: 0.61 (chloroform/methanol, 9:1); FT-IR (ATR, cm^−1^) 3675, 3323 (NH), 2988, 2976, 2901 (Ar-C-H), 1709 (C=O), 1598, 1587 (C=N), 1512, 1450 (C=C), 1240 (C-O-C); ^1^H NMR(400 MHz, DMSO-*d*_6_) *δ* ppm 14.11 (s, 2H, NH), 10.18 (s, 2H, iminic), 8.256–8.249 (d, 2H, *J* = 2.8 Hz, pyridinic), 8.198–8.176 (t, 1H, *J* = 8.8 Hz, pyridinic), 7.88–7.84 (m, 4H, arylic), 7.12–7.08 (m, 4H, arylic); 3.83 (s, 6H, OCH_3_); ^13^C NMR (100 MHz, DMSO-*d*_6_) *δ* ppm 163.19, 162.51, 161.56, 152.38, 149.50, 139.46, 130.71, 124.46, 118.01, 114.66, 55.86.

#### 3.3.2. Synthesis of 6-Metal Complexes **7a**–**e**

The metal complexes **7a**–**e** were synthesized by refluxing the metal chloride salts (1 m.molar) with that of ligand 6 (1 m.molar, 15.34 mg) in ethanol (30 mL). The colored metal complexes of the ligand were afforded with reasonable yield, which were separated by removing the solvent under reduced pressure and dried in a vacuum desiccator with silica blue as dehydrating agents (Supporting Information Figures S5–S8).

##### Ligand–Fe(II) Complex (**7a**)

Reddish powder; yield: 66%; 2.5 g; mp: 218–220 °C; R_f_: 0.59 (chloroform/methanol, 9:1); FT-IR (ATR, cm^−1^) 3675, 3531, 3182 (NH), 3071, 3003, 2989, 2934, 2910, 2834 (Ar-C-H), 1700 (C=O), 1657, 1609 (C=N), 1596, 1586, 1572, 1512 (C=C), 1240 (C-O-C).

##### Ligand–Co(II) Complex (**7b**)

Greenish powder; yield: 66%; 2.3 g; mp: 231–233 °C; R_f_: 0.60 (chloroform/methanol, 9:1); FT-IR (ATR, cm^−1^) 3523, 3390 (NH), 3175 (Ar-C-H), 1712 (C=O), 1609 (C=N), 1504, 1456, 1424 (C=C), 1251 (C-O-C).

##### Ligand–Ni(II) Complex (**7c**)

Brick red powder; yield: 67%; 2.5 g; mp: 234–236 °C; R_f_: 0.58 (chloroform/methanol, 9:1); FT-IR (ATR, cm^−1^) 3675, 3323 (NH), 2988, 2976, 2934, 2901 (Ar-C-H), 1709 (C=O), 1598, 1587 (C=N), 1455, 1450, 1411 (C=C), 1238 (C-O-C).

##### Ligand–Cu(II) Complex (**7d**)

Mustard yellow powder; yield: 67%; 2.8 g; mp: 228–230 °C; R_f_: 0.58 (chloroform/methanol, 9:1); FT-IR (ATR, cm^−1^) 3675, 3323 (NH), 3073, 2988, 2901 (Ar-C-H), 1675 (C=O), 1602, 1581 (C=N), 1554, 1498, 1453, 1418 (C=C), 1291 (C-O-C).

##### Ligand–Zn(II) Complex (**7e**)

Gray powder; yield: 67%; 2.5 g; mp: 216–218 °C; R_f_: 0.59 (chloroform/methanol, 9:1); FT-IR (ATR, cm^−1^) 3445, 3372 (NH), 2988, 2978, 2901 (Ar-C-H), 1698 (C=O), 1601, 1577 (C=N), 1510, 1495, 1471, 1460, 1440, 1420 (C=C), 1259 (C-O-C).

### 3.4. Spectroscopic Analysis

The 1 mM ligand and its metal complex stock solution were prepared in tetrahydrofuran with a total volume of 10 mL, and optical analysis was carried out using a 3 mL cuvette. The measuring concentration of the test solution was kept at 100 µM in the 3 mL cuvette. All the spectral recordings were performed at ambient temperature, and the test samples were shaken well before the operation to ensure uniformity [46,47,48]. The spectral recording for the sample was conducted in triplicate, and the range of the measurement was kept at 200–900 nm.

### 3.5. Enzyme Inhibition

The indophenol method as outlined by Weatherburn [49] was employed to assess urease activity by measuring the produced ammonia. Briefly, incubation of the reaction mixture was carried out at 37 degrees Celsius for half an hour on the microplate reader with 96-well plates. The operated mixture consisted of 20 microliters of the enzyme (from 5 units per ml of stock solution), 20 µL of the tested compound (5 mg/mL stock solution), and 50 microliters of the buffer solution. Thiourea was employed as a reference inhibitor, and percentage inhibition was calculated by dividing the optical density of the sample by the control.

### 3.6. Enzyme Kinetics

The most active compounds, **7c** and **7d**, were found to exhibit different types of inhibition responses. The extent of urea inhibition was assessed by treating them with varying concentrations of these compounds (0, 6.25, 12.5, 25, and 50 µM). Slope versus inhibitor concentration was utilized to find out the Ki, while Ki′ was calculated by plotting inhibitor concentration versus inverse of V via a secondary replot. For urease kinetics experiments, the concentration of substrate was briefly adjusted from 3.12 to 100 mM, and the other steps were carried out according to the protocol employed to track the inhibitory response of urease. Ammonia measurements were employed to determine urease activity following the reported procedure [50].

### 3.7. Docking Analysis

The Autodock 4.2 Vina software (the Scripps Institute) using the Lamarckian genetic algorithm was utilized for docking analysis. The RCSB protein databank (PDB ID: 3LA4) was employed for the evaluation of Jack Bean urease crystal structure. The protein preparation was conducted using Autodock 4 tools, and all water molecules and in-bound ligands and heteroatoms were deleted; furthermore, polar hydrogens and Kollman were assigned, and Gasteiger charges were computed. The structures of the ligands (**7b**, **7c**, and **7d**) were sketched on ChemDraw and converted to the pdb format by PyMoL. After the preparation of protein and ligand molecules, the Autodock Vina run utilized G.A. with a population size of 150 and maximal energetics of 2.5 megagenerations. The grid spacing of 25 Å, with the centroid coordinates of x = −39.87, y = −43.74, and z = 68.37, was employed for the receptor grid generations towards the urease catalytic site. The binding energy was obtained for the best conformational pose, and the interactions were analyzed by BIOVIA Discovery Studio 3.5 [51].

## 4. Conclusions

Herein, we describe the synthesis of a symmetrical molecule via Schiff base formation, which was subsequently complexed with selected transition metals to investigate its UV/fluorescence response, inhibition potential against urease, and molecular docking interactions. Testing of the symmetrical cage-like ligand and its complexes with various eco-friendly transition metals revealed notable urease inhibition activity. The ligand itself displayed an IC_50_ value of 21.80 ± 1.88 µM, similar to thiourea. Interestingly, when complexed with nickel and copper, the ligand’s potency significantly increased, yielding IC_50_ values of 11.8 ± 1.14 and 9.31 ± 1.31 µM, respectively. The cobalt complex showed urease inhibition similar to that of thiourea, while the zinc and iron complexes were less effective, possibly due to weaker interactions with the target protein. Additionally, all metal complexes exhibited strong optical properties, indicating their potential use in fluorescence-guided drug delivery. Molecular docking results, along with IC_50_ values from in vitro urease inhibition assays, indicated a trend of increasing activity among the synthesized compounds, with compound **7d** being the most potent, followed by **7c** and **7b**. Enzyme kinetics analysis was investigated by the Lineweaver–Burk plot, which showed mixed as well as non-competitive modes of inhibition for the most active molecules, i.e., **7c** and **7d**.

## Figures and Tables

**Table 1:** Analysis of the synthesized molecule **6** and its metal complexes **7a**–**e**.

Comp.	^a^ App.	^b^ MP	^c^ R_f_	FT-IR					
				-N-H	Ar-C-H	C=O	C=N	C=C	C-O-C
**6**	Yellow	181–183	0.61	3675, 3323	2988, 2976, 2901	1709	1598, 1587	1512, 1450	1240
**7a** [6-Fe(II)]	Reddish	218–220	0.59	3675, 3531, 3182	3071, 3003, 2989, 2934, 2910, 2834	1700	1657, 1609	1596, 1586, 1572, 1512	1240
**7b** [6-Co(II)]	Greenish	231–233	0.60	3523, 3390	3175	1712	1609	1504, 1456, 1424	1251
**7c** [6-Ni(II)]	Brick red	234–236	0.58	3675, 3323	2988, 2976, 2934, 2901	1709	1598, 1587	1455, 1450, 1411	1238
**7d** [6-Cu(II)]	Mustard yellow	228–230	0.58	3675	3073, 2988, 2901	1675	1602, 1581	1554, 1498, 1453, 1418	1291
**7e** [6-Zn(II)]	Gray	216–218	0.59	3445, 3372	2988, 2978, 2901	1698	1601, 1577	1510, 1495, 1471, 1460, 1440, 1420	1259

^a^ Physical appearance; ^b^ melting point; ^c^ retardation factor values/mobile phase: chloroform/methanol, 9:1.

**Table 2:** The absorption spectral data of 6 and its respective complexes.

Sample	Electronic Transitions			
	π→π*		n→π*	
	λ_max_ (nm)	Molar Absorptivity (M^−1^cm^−1^)	λ_max_ (nm)	Molar Absorptivity (M^−1^cm^−1^)
**6**	241	0.84 × 10^4^	291	0.53 × 10^4^
**7a** [6-Fe(II)]	242	0.95 × 10^4^	299	0.134 × 10^5^
**7b** [6-Co(II)]	244, 289	0.952 × 10^4^; 0.607 × 10^4^	654	0.396 × 10^4^
**7c** [6-Ni(II)]	243, 289	0.91 × 10^4^; 0.55 × 10^4^	342	0.147 × 10^4^
**7d** [6-Cu(II)]	241, 304	0.99 × 10^4^; 0.156 × 10^5^	841	0.44 × 10^4^
**7e** [6-Zn(II)]	241	0.898 × 10^4^	296	0.10 × 10^5^

**Table 3:** Solvent effect on the optical properties of ligands and their metal complexes.

Comp.	Solvents							
	THF		Ethanol		DMSO		Acetonitrile	
	π→π*	n→π*	π→π*	n→π*	π→π*	n→π*	π→π*	n→π*
**6**	0.89	0.53	0.891	0.53	0.88	0.53	0.89	0.54
**7a** [6-Fe(II)]	0.95	1.34	0.95	1.35	0.94	1.33	0.96	1.35
**7b** [6-Co(II)]	0.952, 0.607	0.396	0.953, 0.607	0.396	0.951, 0.607	0.39	0.955, 0.61	0.399
**7c** [6-Ni(II)]	0.91, 0.55	0.147	0.92, 0.56	0.147	0.90, 0.52	0.144	0.92, 0.55	0.147
**7d** [6-Cu(II)]	0.99, 1.56	0.44	0.99, 1.57	0.44	0.96, 1.53	0.43	0.98, 1.58	0.47
**7e** [6-Zn(II)]	0.898	01	0.898	1.1	0.891	0.9	0.899	1.1

**Table 4:** The results of fluorescence analysis for **6** and **7a**–**e**.

Sample	Emission Spectral Analysis			
	^1^ λ_max_Em (nm)	^2^ λ_max_Ex (nm)	Signal Intensity (a.u.)	^3^ ΦFl
**6**	614	350	3482	0.09
**7a** [6-Fe(II)]	643	350	15,253	0.14
**7b** [6-Co(II)]	621	350	19,270	0.18
**7c** [6-Ni(II)]	617	350	14,867	0.15
**7d** [6-Cu(II)]	601	350	13,863	0.15
**7e** [6-Zn(II)]	623	350	17,185	0.17

^1^ Emission maxima; ^2^ excitation for emission measurement; ^3^ relative fluorescence quantum found using rhodamine B as reference.

**Table 5:** Recently reported triazole-based scaffolds and their metal complexes as urease inhibitors.

S. No.	Molecules	IC_50_ (µM)	Reference Drug	Ref.
**1**	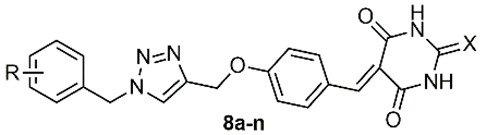	76.53 ± 0.46 to 08.10 ± 0.17	Thiourea/IC_50_ (µM) 22.0 ± 0.03	[35]
**2**	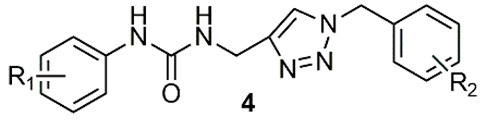	184.43.10 ± 2.16 to 22.81 ± 0.05	Hydroxyurea and thiourea/IC_50_ (µM); 100 ± 0.15 and 23 ± 1.7	[36]
**3**	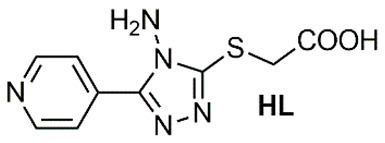	15.094 ± 2.218 (HL) 4.052 ± 0.693 (HLZn) 6.868 ± 1.006 (HLFe)	Acetohydroxamic acid/IC_50_ (µM); 7.898 ± 0.898	[37]
**4**	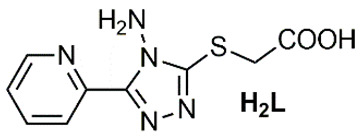	14.7 ± 1.4 (H_2_L) 3.5 ± 0.9 (H_2_LCo(II)) 1.3 ± 0.7 (H_2_LNi(II))	Acetohydroxamic acid/IC_50_ (µM); 7.898 ± 0.898	[38]
**5**	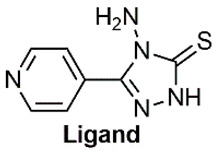	>100 (ligand) 48.16 ± 2.46 to 15.22 ± 1.57 (ligand + Ni(II)	Acetohydroxamic acid/IC_50_ 32.09 ± 5.09	[39]

**Table 6:** The inhibitory effect of synthesized compound **6** and its metal complexes towards urease.

Compounds	Urease Inhibition IC_50_ (µM)	Compounds	Urease Inhibition IC_50_ (µM)
**6**	21.80 ± 1.88	**7c** [6-Ni(II)]	11.82 ± 1.14
**7a** [6-Fe(II)]	31.58 ± 1.71	**7d** [6-Cu(II)]	09.31 ± 1.31
**7b** [6-Co(II)]	22.31 ± 1.90	**7e** [6-Zn(II)]	51.06 ± 4.23
Thiourea/reference drug		Urease inhibition IC_50_ (µM)/20.7 ± 0.45	

## Data Availability

The original contributions presented in the study are included in the article and supplementary material, further inquiries can be directed to the corresponding authors.

## Supplementary Materials

The following supporting information can be downloaded at https://www.mdpi.com/article/10.3390/molecules29204899/s1.
