# Supplementary material for: Synthesis, Urease Inhibition, Molecular Docking, and Optical Analysis of a Symmetrical Schiff Base and Its Selected Metal Complexes
Source: Molecules. 2024 Oct 16;29(20):4899. doi: 10.3390/molecules29204899 (PMC11510561; doi:10.3390/molecules29204899)
Supplement: Supplementary file 1 [file molecules-29-04899-s001.zip › molecules-3223158-supplementary.pdf]

Synthesis, Urease Inhibition, Molecular Docking and Optical Analysis of Symmetrical Schiff Base and its Selected Metal Complexes

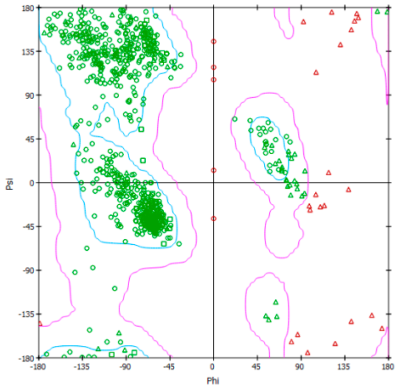

Fig. S1. Ramachandran plot of 3LA4

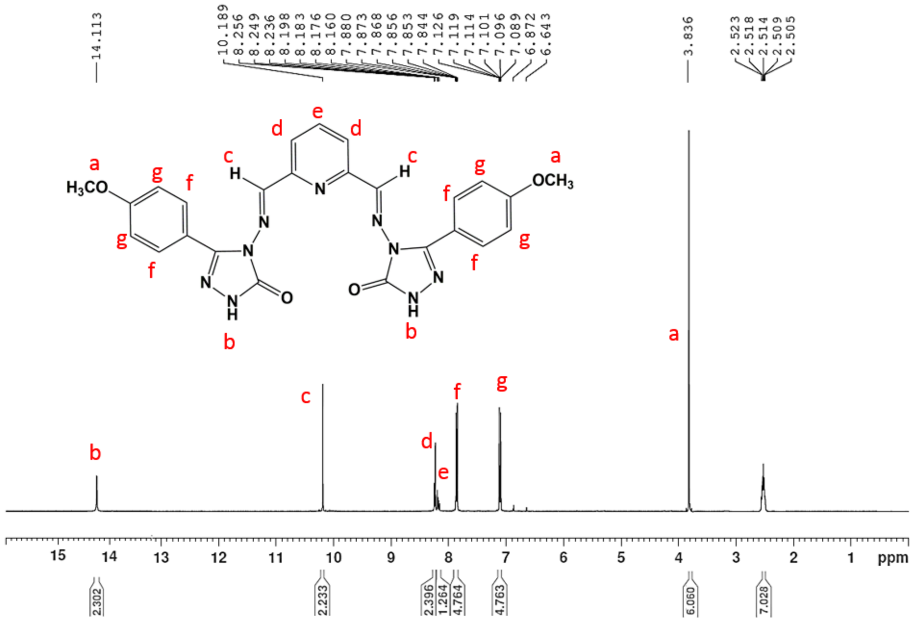

Fig. S2 ^{1}H NMR of ligand 6

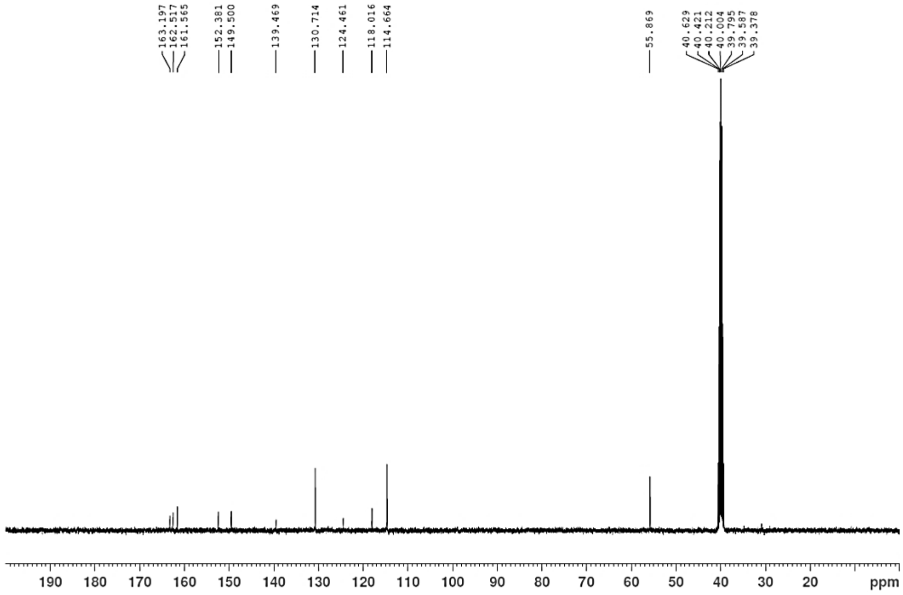

Fig. S3 ^{13}C NMR of ligand 6

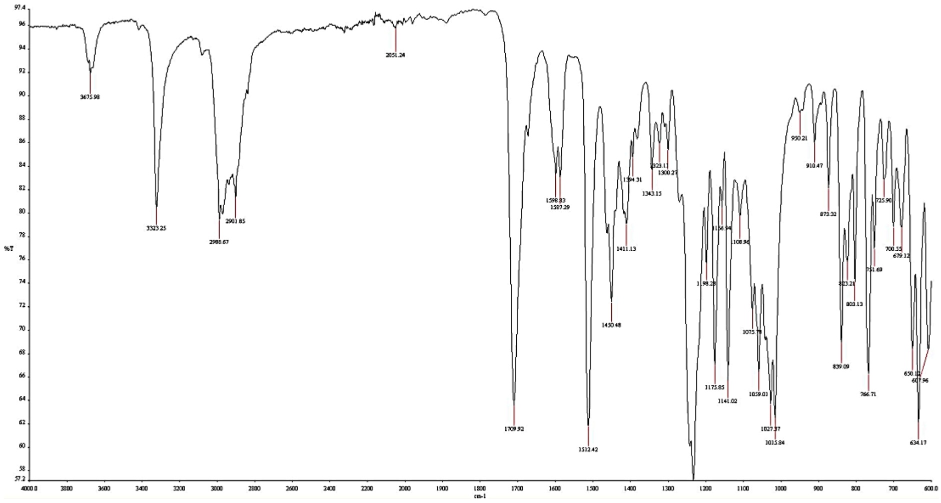

Fig. S4 FT-IR of ligand 6

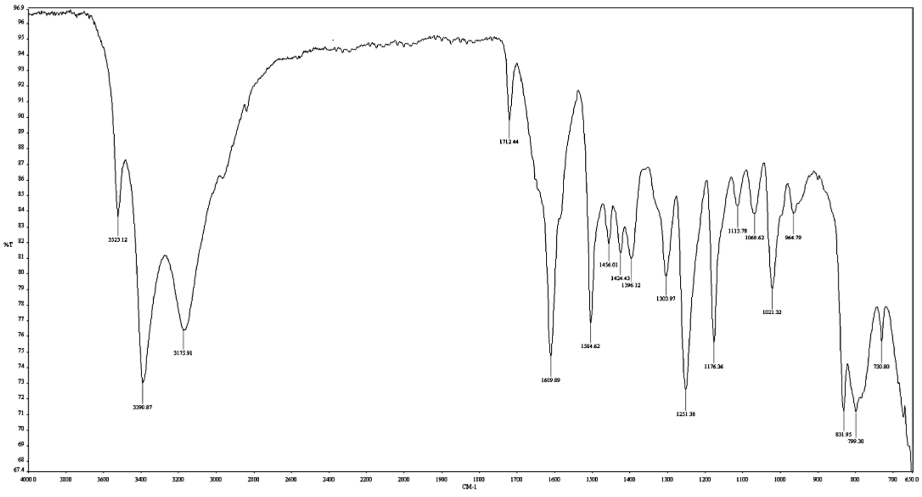

Fig. S5 FT-IR of ligand 6-Co(II)

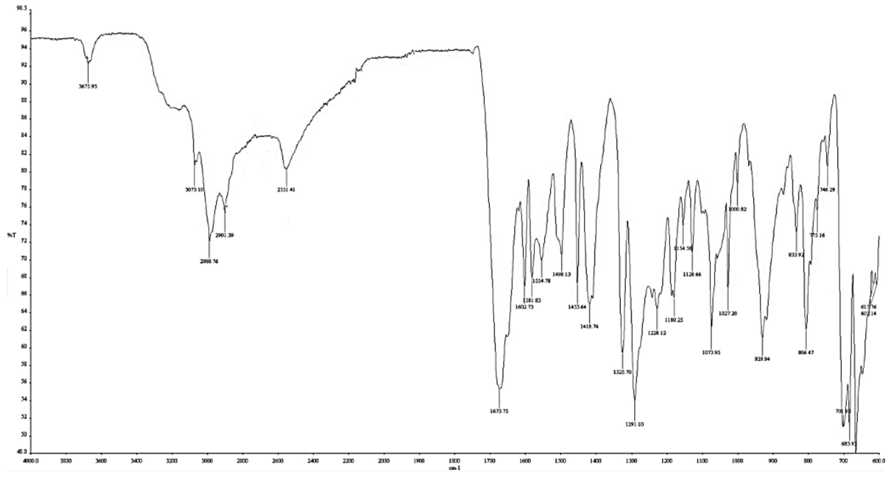

Fig. S6 FT-IR of ligand 6-Cu(II)

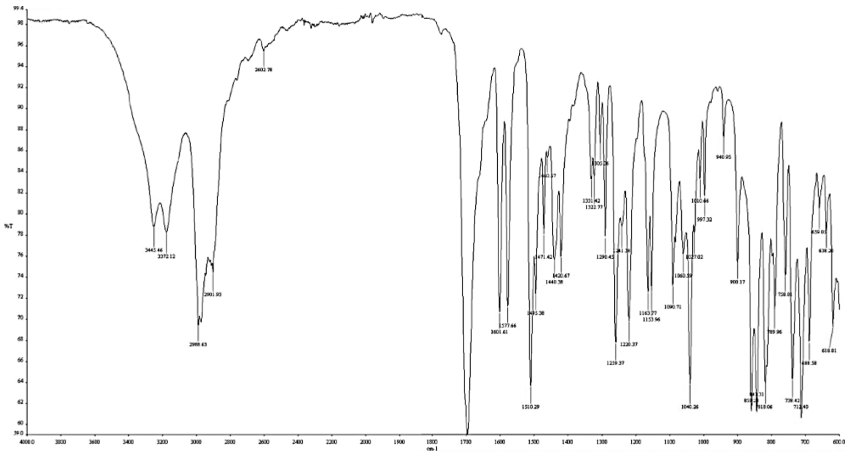

Fig. S7 FT-IR of ligand 6-Zn(II)

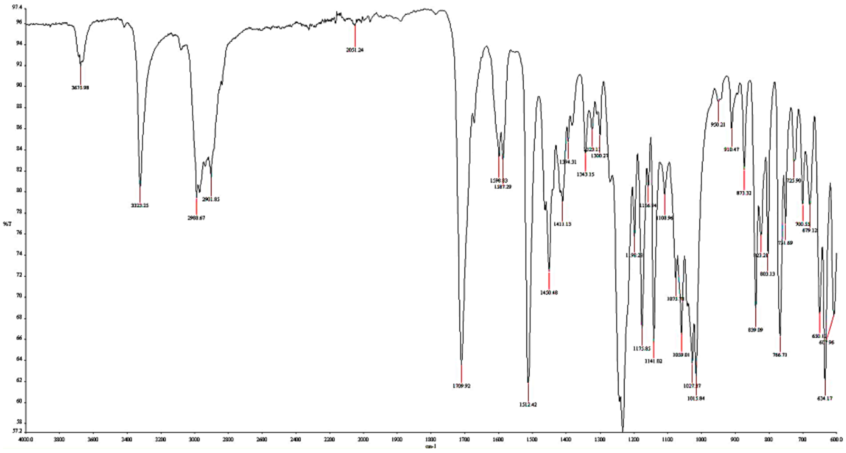

Fig. S8 FT-IR of ligand 6-Ni(II)
